# Erythrocytes morphology and hemorheology in severe bacterial infection

**DOI:** 10.1590/0074-02760190326

**Published:** 2019-12-20

**Authors:** Ayrles FB Silva, Jeanlex S Sousa, Pablyana LR Cunha, José V Lima-Filho, Nylane MN Alencar, Cleverson DT Freitas, Claudio LN Oliveira, Marcio V Ramos

**Affiliations:** 1 Universidade Federal do Ceará, Departamento de Bioquímica e Biologia Molecular, Fortaleza, CE, Brasil; 2 Universidade Federal do Ceará, Departamento de Física, Fortaleza, CE, Brasil; 3 Universidade Federal do Ceará, Departamento de Química Orgânica e Inorgânica, Fortaleza, CE, Brasil; 4 Universidade Federal Rural de Pernambuco, Departamento de Biologia, Recife, PE, Brasil; 5 Universidade Federal do Ceará, Departamento de Fisiologia e Farmacologia, Fortaleza, CE, Brasil

**Keywords:** atomic force microscopy, blood, red blood cells, *Salmonella* Typhimurium

## Abstract

**BACKGROUND:**

Severe bacterial infections initiate inadequate inflammation that leads to disseminated intravascular coagulation and death.

**OBJECTIVES:**

To evaluate the influence of bacterial infection on blood viscosity and red blood cells (RBCs) morphology, and the ability of *Calotropis procera* proteins (CpLP) to prevent the patho-hemorheology in infected animals.

**METHODS:**

Rheology of blood, atomic force microscopy measurements on specific blood elements and blood count were performed to examine changes in blood viscosity, RBCs morphology, platelets activation, and RBCs indices.

**FINDINGS:**

Infected mice hold their blood rheological behaviour as compared to that of the control group. However, they presented hyperactivated platelets, RBCs at different stages of eryptosis, and variation on RBCs indices. CpLP administration in healthy animals altered blood behaviour from pseudoplastic to Bingham-like fluid. Such effect disappeared over time and by inhibiting its proteases. No alterations were observed in RBCs morphology or platelets. Treatment of infected animals with CpLP prevented the changes in RBCs indices and morphology.

**MAIN CONCLUSIONS:**

The inflammatory process triggered by bacterial infection induced pathological changes in RBCs and platelets activation. Treatment of infected animals with CpLP prevented the emergence of RBCs abnormal morphology and this may have implications in the protective effect of CpLP, avoiding animal death.

Gram-negative bacterial infections may provoke systemic inflammation as consequence of bacterial spread to different sites through the bloodstream. This is frequently observed in patients with depressed immune system and economically affects the health system of different countries. The crosstalk between infection, inflammation and coagulation system is well described in the literature.^1^ ^,^ ^2^ The infection-associated activation of coagulation involve several pathways, including activation of endothelial cells to a procoagulant state, impairment of coagulation inhibitory mechanism and suppression of fibrinolytic system.^1^ ^,^ ^3^ On the other hand, peptidases resulting from the coagulation pathway, such as thrombin and activated protein C, enhance the inflammatory process.^4^ Thus, while the acute inflammation associated to bacterial infections affects the coagulation pathways, activated coagulation also affects the inflammatory response.^3^ ^,^ ^5^

In addition, studies have reported that the multifactorial inflammation process affects blood rheology, changing parameters such as whole blood viscosity, platelet aggregation, and erythrocyte aggregation and pathologic deformability.^6^ ^,^ ^7^ ^,^ ^8^ During infection, endotoxin and pro-inflammatory molecules increase platelets reactivity resulting in activation and aggregation.^3^ Meanwhile, red blood cells (RBCs) membrane interacts with the inflammatory molecules which may lead to RBC pathological deformability and eryptosis, the process of RBC cell death.^6^

Eryptosis is defined as the suicidal erythrocyte death that enables the clearance of defective and infected blood cells. It is triggered by a wide number of inductors, including inflammatory molecules and bacterial sphingomyelinase and hemolysin.^9^ ^,^ ^10^ Eryptosis phenomenon progresses by cell shrinkage, membrane deformities and structural disorders into lipid bilayer. The last one may occur due to induction of membrane phospholipid redistribution with the concentration of phosphatidylserine in the outer cell membrane.^10^ This process induces the macrophage engulfment of the eryptotic cell, the release of pro-inflammatory cytokines, and the blood clotting.^9^ ^,^ ^10^

The mouse model of infection with non-typhoidal *Salmonella enterica* serovar Typhimurium (STM) was taken here as a study-model. This bacterium is the preferred organism to study systemic bacterial infection.^11^ STM can grow in the intracellular environment and manipulate many biological processes within the host. It induces inflammation and exploits this process as a tool to overcome the competition with the resident microbiota in the gut and to enhance its colonisation and replication.^11^ ^,^ ^12^ This bacterium elicits a self-limiting gastroenteritis and it is an important food born pathogen.^13^ ^,^ ^14^ Here it is further characterised the effects of STM infection on blood viscosity and on topographical features of RBCs and platelets.

In order to minimise the damage caused on cell morphology and alterations on blood rheology, observed in animals undergoing severe bacterial infection, therapeutic agents capable of control the inflammatory process associated to this condition should be investigated. To this goal, *Calotropis procera* latex proteins (CpLP), that were previously shown to modulate inadequate inflammatory processes, were used in this study. It was hypothesised whether CpLP would positively influence blood viscosity and reduce both the inflammatory damage in RBCs and the hyperactivation of platelets during bacterial infection. Studies have demonstrated that this latex exhibit antagonistic anti-inflammatory and inflammatory activities depending on fraction used and route of administration.^15^ The effect of CpLP on the protection of mice infected with STM was previously demonstrated.^16^ ^,^ ^17^ Animals severely infected by STM overcome all the adverse physiological effects resulting from the bacteria activity and death is not observed when CpLP is administered to them.

Related to the coagulation process, CpLP exhibit procoagulant properties. It accelerates the coagulation cascade by activation of the intrinsic pathway and reduces the clotting time of the plasma.^18^ In addition, the subfractions of CpLP possess proteolytic activity with both thrombin- and plasmin-like effects.^18^ Alterations on erythrocytes morphology and hemorheology are certainly pivotal events contributing to the disseminated intravascular coagulation phenomenon, the ultimate step of the physiological collapse and death. It is therefore important to get new insights in this physiological event and in the understanding of how latex proteins are efficient in preventing death in animals severely infected.

## MATERIALS AND METHODS

*Animals and ethical aspects* - Adult male Swiss mice weighing approximately 30 g were used in this study. The animals were obtained from the Biotery of the Universidade de Fortaleza and kept in an animal house with controlled lighting (12-h light-dark cycles), temperature (25ºC) and humidity (60-70%), with free access to water and commercial sterile diet. The study was approved by the Ethics Committee on Animal Research of the Universidade Federal do Ceará, under permission No. 8604181217 and was performed in accordance with the guidelines issued by the National Council for Control of Animal Experimentation (CONCEA).

*Bacterial strain and infection* - For experimentation, it was used the mouse virulent *Salmonella enterica* serovar Typhimurium (strain C5). The bacteria were activated in Brain Heart Infusion broth at 37ºC for 18 h and then cultured in BHI agar for another 24 h at 37ºC. Colony-forming units (CFU) were diluted in sterile saline to attain a bacterial suspension containing approximately 10^8^ CFU/mL (according to the 0.5 tube of the McFarland scale). The bacterial suspension was diluted 10-fold, and 0.2 mL (10^7^ CFU/mL) was administered intraperitoneally in the animals. Serial dilutions of the remaining bacterial solutions were plated onto MacConkey agar plates to determine the exact bacterial CFU used.

*Latex and laticifer proteins* - The latex of *C. procera* (Ait.) R. Br. (Asclepiadaceae) was collected from the terminal branches of healthy plants growing in Fortaleza, Ceará, Brazil. The voucher (sample specimen no. 32663) was deposited at the Prisco Bezerra Herbarium of the Universidade Federal do Ceará. The access and use of this biological resource were performed after registration and legal authorisation according to the current Brazilian law for biodiversity (Agreement n. A689147). A small incision was made and the latex was left to flow off into distilled water in order to obtain a mixture 1:1 (v/v). Soluble proteins (latex proteins from *C. procera*, CpLP) were recovered from the whole latex according to the method previously described.^15^ To inhibit the endogenous proteolytic activity present in this fraction, CpLP was treated with 30 mM iodoacetamide (IAA) and this sample was named CpLP + IAA, as described.^19^ All further experiments were performed using the lyophilised material diluted in saline.

*Experimental design* - To evaluate the effect of STM infection on erythrocytes morphology, hemorheology, and RBCs indices and to analyse the consequence of CpLP treatment in infected mice, the experimental procedure was performed as previously described.^18^ Briefly, the animals were initially divided into four groups each comprising six mice. Group I served as a control where 0.2 mL of sterile saline was administered intraperitoneally. Group II, named CpLP, were animals that received CpLP (30 mg/kg) intraperitoneally. The systemic infection was induced in group III and group IV by intraperitoneal administration of bacterial suspension (10^7^ CFU/mL). Thus, group III was named *S*. Typhimurium. The effect of CpLP (30mg/kg) during the STM infection was studied in group IV. In this group, the animals were treated with CpLP 24 h before bacterial inoculation. The protein dose was based on previous studies using CpLP in the treatment of STM infection.^16^ ^,^ ^17^

The blood was collected 24 h after saline or CpLP administration or 24 h after the bacterial challenge, from the retroorbital plexus of the anesthetised mice. The early time point was defined based on previous studies. These demonstrated that infected animals come to death within three days of infection.^17^ Furthermore, within 24 h there was a clear difference between heath and infected animals, related to clotting time and number of platelets.^18^ All these parameters are related to the homeostasis of the blood system and these former results justify the time-course applied in the present study.

To elucidate the transient influence of CpLP in viscosity, animals treated as described in group II were sacrificed 48 and 72 h after proteins administration and its blood was analysed as followed described. Furthermore, one more group was added to analyse the influence of the peptidases present in CpLP on the increased viscosity observed in mice. Thus, CpLP + IAA (30 mg/kg) was intraperitoneally administrated in mice and the blood viscosity was analysed 24 h after proteins administration.

*Rheology* - The blood rheology was analysed using a stress-controlled rheometer (TA Instruments Rheometer, model AR-550) equipped with a temperature controller. A circular parallel plate cell with internal diameter of 25 mm and a height of 28 mm, which is the gap between plates, was employed in the experiment. The equipment was configured to get 20 points within the shear rate between 0.1 - 10 Pascal. All rheology experiments were conducted at 25ºC. To investigate different hypotheses, data were obtained from three independent experiments, namely, the effect of infection in the whole blood rheology, the effect of CpLP administration in healthy animals and the effect of CpLP treatment to the infected group. In order to determine rheological parameters of the whole blood in all those different conditions and experiments performed in this work, the rheogram data were fitted to the well-known Bingham fluid behaviour,

$$\tau =\tau 0\;+k\gamma^{n},\;(1)$$

$$µ\;=\;\tau 0/\;\gamma \;+\;k\gamma^{n-1},\;(2)$$

where τ is the measured shear stress, γ is the applied shear rate, k is the rheological consistency index, n is a dimensionless flow behaviour index, µ is the viscosity, and τ_0_ is the yield stress value. Fluids exhibiting non-zero yield stress behave as a rigid body at low stresses but flow as a viscous fluid at high stresses. For τ_0_ = 0, however, such behaviour is no longer observed and the fluid presents a special case of Bingham model where the exponent n determines the kind of the fluid, i.e., Newtonian fluid for the linear behaviour (n = 1) and non-Newtonian otherwise. In the latter case, viscosity changes according to shear rate experienced by the fluid. Moreover, non-Newtonian fluids can be classified as shear thickening, also called dilatant, for n > 1, or shear thinning, also called pseudoplastic, for n < 1. As it is shown later, blood usually behaves as pseudoplastic fluid (when τ_0_ = 0 and n < 1) but the presence of certain proteins can change the blood behaviour to the more general case of Bingham fluids (when τ_0_ > 0).

*Atomic force microscopy imaging* - Blood samples collected were used to perform monolayers of blood on glass slides, dried in air for thirty minutes. No chemical ﬁxators were used. The images of the platelets, cells and RBCs membrane surface were obtained using AFM (MFP-3D, Asylum-Research, Santa Barbara, CA, USA) in intermittent contact mode. Standard silicon cantilevers (NCHR, Nano World) with nominal spring constant of 42 N/m were used. All images were obtained within three hours after blood collection.

*Hematological parameters* - A semiautomatic cellular analyser Sysmex KX-21 N (Roche, USA) was used to determine the hematological parameters. Red blood cell count (RBC [10^6^ */*μL]), hemoglobin concentration (Hgb [g/dL]), hematocrit (Hct [%]), mean corpuscular volume (MCV [fL]), mean corpuscular hemoglobin (MCH [pg]), mean corpuscular hemoglobin concentration (MCHC [g/dL]) and platelet count (Plt [10^3^ */*μL]) are presented in this paper.

*Statistical analysis* - To verify if the treatment had significant effect on the viscosity, experimental groups (*S.* Typhimurium, CpLP, and CpLP + *S.* Typhimurium) were compared with the control group (saline) using the One-way analysis of variance test, followed by Bonferroni’s test. Differences were considered statistically significant when p-value ≤ 0.05.

## RESULTS

**Fig. 1:**
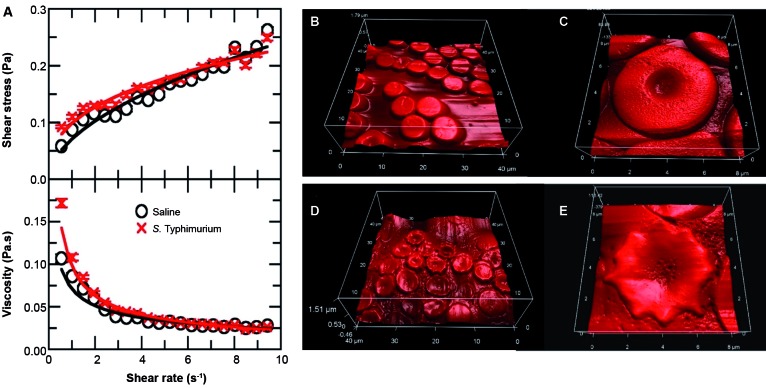
effects of bacterial infection on whole blood viscosity and red blood cells (RBCs) morphology*.* (A) Rheogram (top) and viscosity curve profile (bottom) of blood from healthy and infected animals. Flow curves in the rheogram illustrate shear stress (τ; Pa) vs shear rate (γ; s^-1^) and viscosity curves illustrate dynamic viscosity (η; Pa*s) vs shear stress (γ; s^-1^). These data (symbols) are then fitted with the Bingham fluid behaviour (solid lines) to calculate blood rheological parameters (shown in Table I). Error bars are smaller than the symbols. (B-C) Atomic force microscopy images of whole blood from healthy mice. (D-E) Atomic force microscopy images of whole blood from animals infected with *Salmonella* Typhimurium (10^7^ CFU / mL) whose blood was analysed 24 h post challenge.

**Fig. 2:**
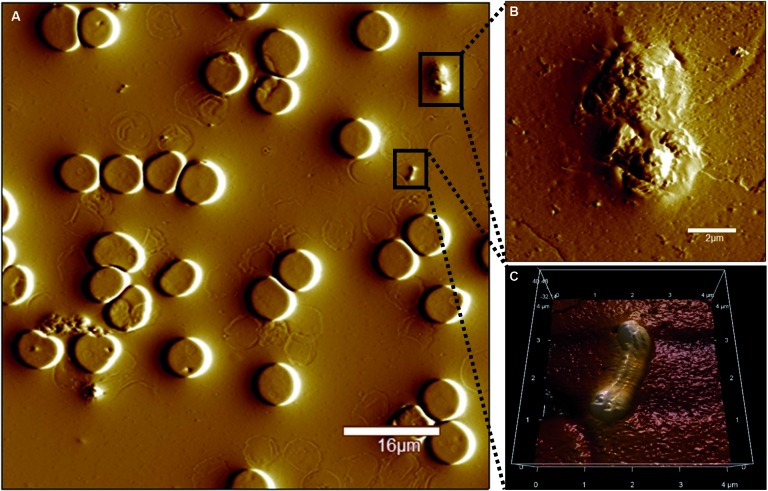
different features observed in the whole blood from infected animals using atomic force microscopy. (A) Whole blood topography micrograph from mice infected with *Salmonella* Typhimurium (10^7^ CFU / mL)*.* (B) Activated platelet with pseudopodia formation. (C) *S.* Typhimurium in the blood of the animal.

*Severe bacterial infection did not increase the whole blood viscosity however altered RBCs morphology and induced platelets activation* - Macro-rheological behaviour of the whole blood, as well as the effect of severe bacterial infection on RBCs morphology and platelets activation were analysed after 24 h of infection. It was observed that mice infected with STM presented RBCs with pathological deformations, such as cell membrane scrambling, cell shrinkage and membrane blebbing or in eryptotic stage (Fig. 1D-E). Under the same condition, it was possible to observe a wide range of hyperactivated platelets with increased pseudopodia formation and aggregation (Fig. 2B) and to confirm the presence of bacteria in the blood (Fig. 2C).

Analysis of the rheogram data showed that the whole blood of infected animals behaves like a pseudoplastic fluid, i.e., the blood rheology obeys equations (1) and (2), with τ_0_ = 0 and n < 1 (Fig. 1A). Yield stress (τ_0_), consistency index (k), and flow behaviour index (n) were obtained as the best values in the fitting of rheogram curves and are displayed in Table I. Interestingly, although the bacterial infection changed drastically the morphology, at micrometric scale, of red blood cells, only a small increase in the viscosity curve was observed within 24 h of study. Overall, the macrometric scale rheology behaviour of the whole blood remains the same.

**Fig. 3:**
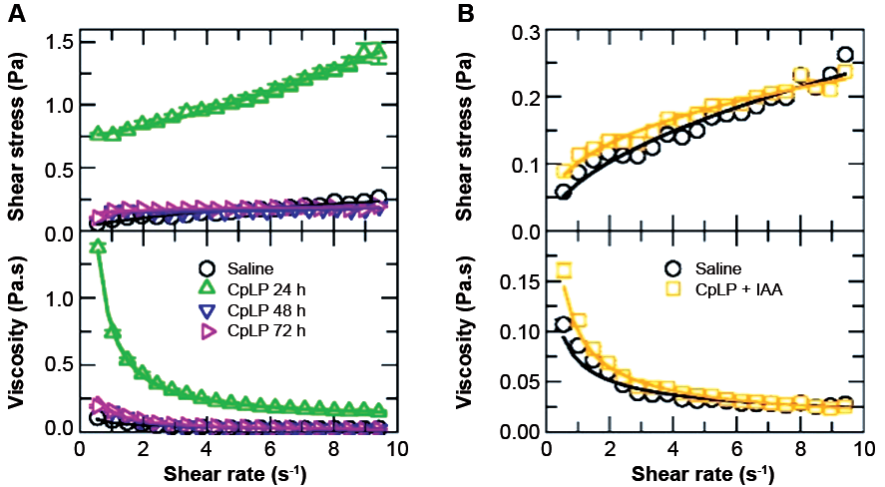
hemorheology of healthy animals administrated with *Calotropis procera* proteins (CpLP). (A) Rheogram (top) and viscosity curve profile (bottom) of blood from mice that received CpLP (30 mg / kg) and whose blood was analysed after different time of exposure. (B) Rheogram (top) and viscosity curve profile (bottom) of blood from healthy animals that received CpLP plus iodoacetamide (30 mg / kg). Flow curves in the rheogram illustrate shear stress (τ; Pa) vs shear rate (γ; s^-1^) and viscosity curves illustrate dynamic viscosity (η; Pa*s) vs shear stress (γ; s^-1^). Solid lines show the respective fit of measured data (shown in symbols) with the Bingham fluid behaviour to calculate blood rheological parameters (shown in Table I). Error bars are smaller than the symbols.

**Fig. 4:**
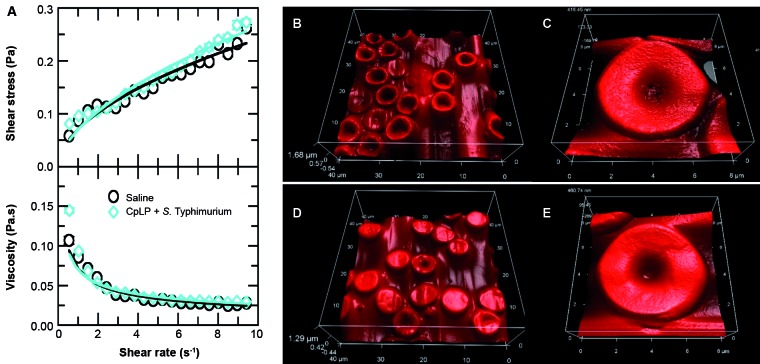
effects of *Calotropis procera* proteins (CpLP) treatment on whole blood viscosity and red blood cells (RBCs) morphology from infected animals*.* (A) Rheogram (top) and viscosity curve profile (bottom) of blood from infected animals treated with CpLP (30 mg / kg). Flow curves in the rheogram illustrate shear stress (τ; Pa) vs shear rate (γ; s^-1^) and viscosity curves illustrate dynamic viscosity (η; Pa*s) vs shear stress (γ; s^-1^). These data (symbols) are fitted with the Bingham fluid behaviour (solid lines) to calculate blood rheological parameters (shown in Table I). Error bars are smaller than the symbols. (B-C) Atomic force microscopy images of whole blood from healthy mice that received CpLP (30 mg / kg) and whose blood was analysed 24 h after exposure. (D-E) Atomic force microscopy views of whole blood from animals pre-treated with CpLP (30 mg / kg) and infected with *Salmonella* Typhimurium. These animals had the blood analysed 24 h after infection.

*CpLP administration to non-infected animals increased the whole blood viscosity due to intrinsic peptidases* - Surprisingly, the administration of CpLP in healthy animals (30 mg/kg) changed the nature of the fluid from pseudoplastic to Bingham fluid after 24 h of administration (see the values of τ_0_ in Table I). In that case, the yield stress is no longer zero and the fluid resists flowing under shear stress less than τ_0_. It means the blood fluidity was reduced. This treatment was considered extremely significant in alter blood viscosity, with p value less than 0.0001 and difference in all shear rate values analysed (Fig. 3A). Such effect, however, changed over time and the blood gradually switched back from Bingham to pseudoplastic after 72 h, as shown by the rheological parameters displayed in Table I. In spite of CpLP changed the whole blood viscosity within 24 h, it had no visible effect on the cells or in the activation of platelets in healthy animals (Fig. 4B-C).

**TABLE I:** Results obtained from data analysis of rheogram curves of blood from mice submitted to different treatments

Groups	Minimum yield stress (τ_0_)	Consistency index (k)	Flow behaviour index (n)
Saline	0.0	0.071	0.53
CpLP - 24 h	0.75	0.031	1.37
CpLP - 48 h	0.15	0.0001	2.46
CpLP - 72 h	0.0	0.150	0.10
CpLP + IAA	0.0	0.100	0.36
*Salmonella* Typhimurium	0.0	0.098	0.37
CpLP + *S.* Typhimurium	0.0	0.070	0.58

CpLP: *Calotropis procera* latex proteins; IAA: iodoacetamide.

In order to understand such phenomenon, we studied the influence of the proteolytic potential of CpLP in this behaviour transition of the whole blood viscosity. Thus, CpLP was treated with IAA to avoid its proteolytic activity. It turned out that the administration of CpLP + IAA in healthy animals had no influence in the blood viscosity and the rheogram demonstrated the regular pseudoplastic behaviour (Fig. 3B).

*Treatment with CpLP prevents erythrocyte abnormalities in infected animals* - Animals previously treated with CpLP (30 mg/kg) and infected with STM did not exhibit any difference in viscosity when compared with the control group and again presented a pseudoplastic behaviour (Fig. 4A). In addition, the animals did not present modified erythrocytes (Fig. 4D-E). In the same way, activated platelets were not widespread when compared with the infected animals. Therefore, CpLP treatment prevented the abnormalities observed in blood cells of animals undergoing infection progress.

*Quantitative hematological parameters* - Haematology of the samples revealed significantly more red blood cells and haematocrit in infected animals compared to the control ones (p < 0.05). In addition, infected mice had lower values of MCV, MCH and platelets in relation to the control group. The previous treatment with CpLP in infected animals prevented all those changes, except platelets count that was increased but not similar to the control group (Table II). Values of MCV and MCH were reduced in animals receiving CpLP, compared to the control ones. Changes observed in these haematological parameters are difficult to interpret at this stage as it would be expected more time to shift since erythrocytes have an estimated lifetime of three months. It is worth noting that CpLP contributed to preserve the blood homeostasis in terms of cellular profile and platelet content in infected animals.

**TABLE II:** Hematological parameters from mice that received different treatments

Groups	Saline	CpLP	*Salmonella* Typhimurium	CpLP + *S.* Typhimurium
RBC (10^6^ */*μL)	9.33 ± 0.08	9.71 ± 0.12	9.91 ± 0.29*	9.05 ± 0.18
HGB, (g/dL)	15.41 ± 0.12	15.28 ± 0.19	15.17 ± 0.48	15.18 ± 0.20
HCT, (%)	43.02 ± 0.33	43.77 ± 0.55	47.78 ± 0.63*	42.20 ± 0.59
MCV, (fL)	46.61 ± 0.32	45.08 ± 0.25*	45.22 ± 0.36*	47.09 ± 0.18
MCH, (pg)	16.38 ± 0.10	15.70 ± 0.07*	15.87 ± 0.13*	16.57 ± 0.12
MCHC, (g/dL)	35.21 ± 0.13	34.93 ± 0.08	35.11 ± 0.22	35.03 ± 0.19
Platelets, (10^3^/μL)	685.0 ± 40.82	770.3 ± 35.93	198.9 ± 45.61*	495.3 ± 44.78*

Blood samples (100 μL) were collected via retroorbital plexus of animals anaesthesiated. All tested samples were given intraperitoneally. Blood samples of Groups “saline”, “CpLP” and “*S*. Typhimurium” were obtained 24 h after administration. Blood samples of animals belonging to group “CpLP + *S*. Typhimurium” were obtained 48 h after CpLP treatment. Data are mean ± scanning electron microscopy (SEM) *p < 0.05 vs. saline group. CpLP: *Calotropis procera* proteins; RBC: red blood cell; HGB: hemoglobin; HCT: hematocrit; MCV: mean corpuscular volume; MCH: mean corpuscular hemoglobin; MCHC: mean corpuscular hemoglobin concentration.

## DISCUSSION

Severe bacterial infections induce an acute inflammatory response in the host. This process has an intricate relationship with the coagulation cascade leading up to microvascular damage and multiple organ failure. Inflammatory molecules are capable of interact with RBCs membrane changing its morphology to a pathological state that eventually result in eryptosis.^3^ ^,^ ^6^ This process influences the hemorheology and it is linked to abnormal coagulation process, one of the hallmarks of inflammation.^6^ Thus, investigating the influence of bacterial infection and its correlated inflammatory process on hemorheological parameters are important to understand blood circulation during the infectious process.

*S.* Typhimurium was the model used in this study due to the accumulated information available about the physiological effects observed in mice undergoing severe infection and the known protective effect of CpLP on animals lethally infected with it. The mechanisms of STM infection and virulence, and the host immune response against this bacterium are extensively studied.^20^ ^,^ ^21^ It has been demonstrated that the mice model of bacterial infection with STM induces an inflammatory process with changing in the levels of biochemical markers of inflammation, such as interleukin-1 (IL-1), interleukin-10 (IL-10), interleukin-12 (IL-12), tumor necrosis factor-α (TNF-α), and nitric oxide^16^ ^,^ ^22^ along the progress of infection. The infection also increases the neutrophils recruitment to the initial locus of infection,^17^ and reduces clotting time and platelet count.^18^ More, it was possible to identify inflammatory infiltrates in the liver and spleen.^16^

Despite of the use of STM to induce inflammatory process be a well-characterised model, here it is demonstrated the influence of this infection in blood rheology, RBCs, and platelets. STM infection did not significantly change the whole blood viscosity within 24 h but it was able to induce inflammation features in the blood system, such as pathological deformations in RBCs, eryptotic RBCs, hyperactivation of platelets, and several alterations in hematological parameters. Abnormal clotting may be induced by raised platelets reactivity and eryptotic RBCs count, with microparticle formation^3^ ^,^ ^5^ which affects the rheology.^23^ Worth pointing out that, not all animals presented RBCs in eryptosis. This may be justified due the relation between severe infection and inflammation that leads up to different stages of hemostatic abnormalities where the severe disseminated intravascular coagulation is the final one.^3^ Also, it should be recorded that during the infection progress, multiple physiological events are sequentially triggered at due times. Even, the pathological state is affected by the intrinsic difference between animals since it has been demonstrated that the virulence and the metabolism in the host are closely related.^11^

In an attempt to revert the patho-hemorheology features observed in infected mice, it was used the richest protein fraction from *C. procera* latex, named CpLP. In a previous study, CpLP has been shown to afford protection in infected mice with regard to the inflammatory response and survival.^16^ The bacterial load in the bloodstream was reduced approximately 100-fold after 24 h of infection, compared with untreated animals, and no significant alterations were seen in liver histology within the same time point.^17^ Further, it was observed that CpLP induced a significant recruitment of neutrophils into the peritoneal cavity, prevented the reduction of lymphocytes in the bloodstream and reduced the level of nitric oxide in serum.^17^ CpLP also significantly reduced the procoagulation and the thrombocytopenia observed in infected mice.^18^

CpLP administration in healthy animals resulted in a change of the kind of fluid that the whole blood is represented, from pseudoplastic to Bingham fluid, increasing the blood viscosity in about one order of magnitude. This increased viscosity is in compliance with the procoagulant effect and the reduced clotting time of the plasma previously observed.^18^ The protein profile of CpLP has been shown and demonstrated the presence of proteolytic enzymes, mainly cysteine peptidases.^24^ ^)^ This kind of enzymes is involved in diverse biological processes and may induce blood coagulation.^18^ ^,^ ^25^ Previously, it was demonstrated that CpLP induces a concentration-dependent decrease in clotting time, accelerates coagulation cascade by intrinsic pathway and induces the formation of fibrin from fibrinogen due to hydrolysis of Aα, Bβ, and γ chains of fibrinogen.^18^ Besides the increased viscosity, no significant alteration was observed in RBCs or platelets under the AFM analyse, suggesting that such effect may be linked to its intrinsic peptidases and not to a possible negative effect in blood elements.

Accordingly, to investigate the relevance of endogenous proteolytic activity in the whole blood viscosity induced by CpLP, treatments with CpLP whose proteolytic activity was inhibited with IAA were conducted. It was demonstrated that CpLP + IAA did not induce any increase in viscosity confirming that this activity is linked to intrinsic peptidases. When CpLP was administrated into healthy animals, without the addition of IAA, the blood presented a high value of yield stress changing its behaviour from pseudoplastic to Bingham-like, within 24 h after CpLP administration. Such effect, however, is time dependent as the blood from animals with CpLP switched back gradually to the normal flow resistance within 72 h. In previous works, the presence of CpLP in healthy animals did not induce toxicity or allergy.^19^ ^,^ ^26^ Thus, it is believed that the influence of peptidases present in the fraction does not interfere in the animal wellbeing.

Samples from infected animals previously treated with CpLP did not present alterations in viscosity. The fact that CpLP increased the viscosity in the moment of bacterial inoculation ought to be considered as a relevant point. This opens the question whether the reduction in bacterial dissemination observed in previous studies^16^ was due to the increased viscosity. Moreover, in the present study, it was not observed morphological changes in RBCs nor widespread activated platelet in those treated animals. In addition, it was measured an increase in platelet count, compared with the infected group what may contribute to CpLP effects. This may be related with the ability of this fraction in reduce inflammatory mediators during STM infection.^16^ ^,^ ^17^

Thus, the present results may improve our understanding of the role of CpLP in the maintenance of blood homeostasis in infected mice. It was also found that bacterial infection leads to extensive modification of erythrocytes and activation of platelets. The blood parameters observed to be altered in this study when the animals were infected or treated with CpLP should be investigated in other models of infection in order to better characterise the preliminary data presented in this study. To the best of our knowledge, this is the first time that atomic force microscopy and rheology are employed together to investigate blood and cellular features in animals experiencing the physiological collapse that anticipates the intravascular disseminated coagulation. Transient increase in blood viscosity due to CpLP administration may be involved in the reduction of STM dissemination. These effects may contribute to prevent abnormal coagulation process and lead to the protection of animals, avoiding death, even suffering severe bacterial infection.

*In conclusion* - The observations reported in this paper therefore point to the consequences of infectious disease in the microcirculation, demonstrating the extensive deformability of erythrocytes and the activation of platelets. Although *Salmonella* Typhimurium deforms red blood cells at micrometric scale, the macroscopic flow behaviour of the blood remains virtually unaltered when compared to the healthy condition. However, such normal bloodstream flow can potentially deliver bacteria all over the body disseminating the infection. Moreover, it is proposed that the protective effect of the latex proteins from *C. procera* may be related to its ability of prevent erythrocytes modifications and hyperactivated platelets impairing the disseminated intravascular coagulation.
